# Association between cardiometabolic index and stroke risk: insights from the CHARLS cohort study

**DOI:** 10.3389/fneur.2025.1548977

**Published:** 2025-07-31

**Authors:** Tian Liu, Chen Xu, Hangjun Chen, Fuhua Chen, Bei Feng, Xiaoying Tao, Yibo Zhou

**Affiliations:** Department of Ultrasound, Affiliated Jinhua Hospital, Zhejiang University School of Medicine, Jinhua, China

**Keywords:** cardiometabolic index, stroke risk, longitudinal study, China Health and Retirement Longitudinal Study (CHARLS), prognostic marker

## Abstract

**Background:**

Stroke is a leading global health challenge, significantly burdening individuals and healthcare systems. The Cardiometabolic Index (CMI), a novel metric combining triglycerides, HDL cholesterol, and waist-to-height ratio, has shown promise in predicting metabolic diseases but its relationship with stroke risk is underexplored.

**Methods:**

This study analyzed data from 9,079 participants in the China Health and Retirement Longitudinal Study (CHARLS). Participants were stratified by CMI quintiles with the following ranges: Q1 (0.074–0.602), Q2 (0.602–0.935), Q3 (0.935–1.402), Q4 (1.402–2.327), and Q5 (2.327–74.326). Stroke incidence over a 7.8-year follow-up period was assessed. Cox proportional hazard models and restricted cubic spline regression were used to evaluate the association between baseline CMI and stroke risk, adjusting for confounders.

**Results:**

Higher CMI levels were significantly associated with increased stroke risk, with a 21% higher risk per 1-unit increase in CMI after adjustment (HR: 1.21, 95% CI: 1.11–1.32). Stroke incidence showed a dose–response relationship, particularly above a CMI threshold of 1.6.

**Conclusion:**

Elevated CMI is a strong independent predictor of stroke risk, highlighting its potential as a clinical tool for early risk stratification and prevention. Further large-scale studies are needed to validate its utility.

## Introduction

Stroke is a major public health issue worldwide, with over 100 million cases and 6.5 million related deaths annually. The high rates of stroke-related disability, including motor impairment, cognitive decline, and death, place a considerable burden on individuals and health systems (1). Given the multifactorial nature of stroke, early identification and management of individuals at risk are essential for effective stroke prevention and improving patient outcomes. Obesity, hypertension, diabetes, and hyperlipidemia are modifiable risk factors that, if managed early, can prevent or delay the onset of stroke (2–4). However, translating this knowledge into practical prevention strategies remains a significant challenge. Therefore, further research into stroke risk factors and the development of effective diagnostic tools is critical to reducing the global stroke burden (5).

The cardiometabolic index (CMI) is a novel metabolism-related index, calculated as the ratio of triglycerides (TG) to high-density lipoprotein cholesterol (HDL-C) multiplied by the waist-to-height ratio (WHtR) (6, 7). Initially developed as a predictor for diabetes risk, the CMI has garnered attention for its association with a variety of health conditions, including cardiovascular disease (CVD), chronic kidney disease (CKD), non-alcoholic fatty liver disease (NAFLD), and metabolic syndrome (MetS) (7–12). Given its strong predictive value for various diseases, further investigation into the CMI’s prognostic capabilities is warranted. Currently, there is a lack of literature documenting the association between CMI and stroke risk. Therefore, our study aimed to evaluate the association of CMI with stroke, utilizing data from the China Health and Retirement Longitudinal Study (CHARLS).

## Methods

### Study populations

CHARLS is an ongoing national cohort investigation designed to assess the social, economic, and health status of individuals aged 45 and older in China (13, 14). The baseline survey, which began in 2011, initially included 17,705 participants from 10,257 households, drawn from 150 counties across 28 provinces. In addition to comprehensive sociodemographic data, the survey also collects biomedical measurements, health status, and functional ability information through standardized questionnaires and physical exams. CHARLS has been conducted in five waves, spanning from 2011 to 2020. The study has been approved by the Biomedical Ethics Review Board of Peking University (IRB00001052-11015), and all participants provided written informed consent before their inclusion in the research.

**Figure 1:**
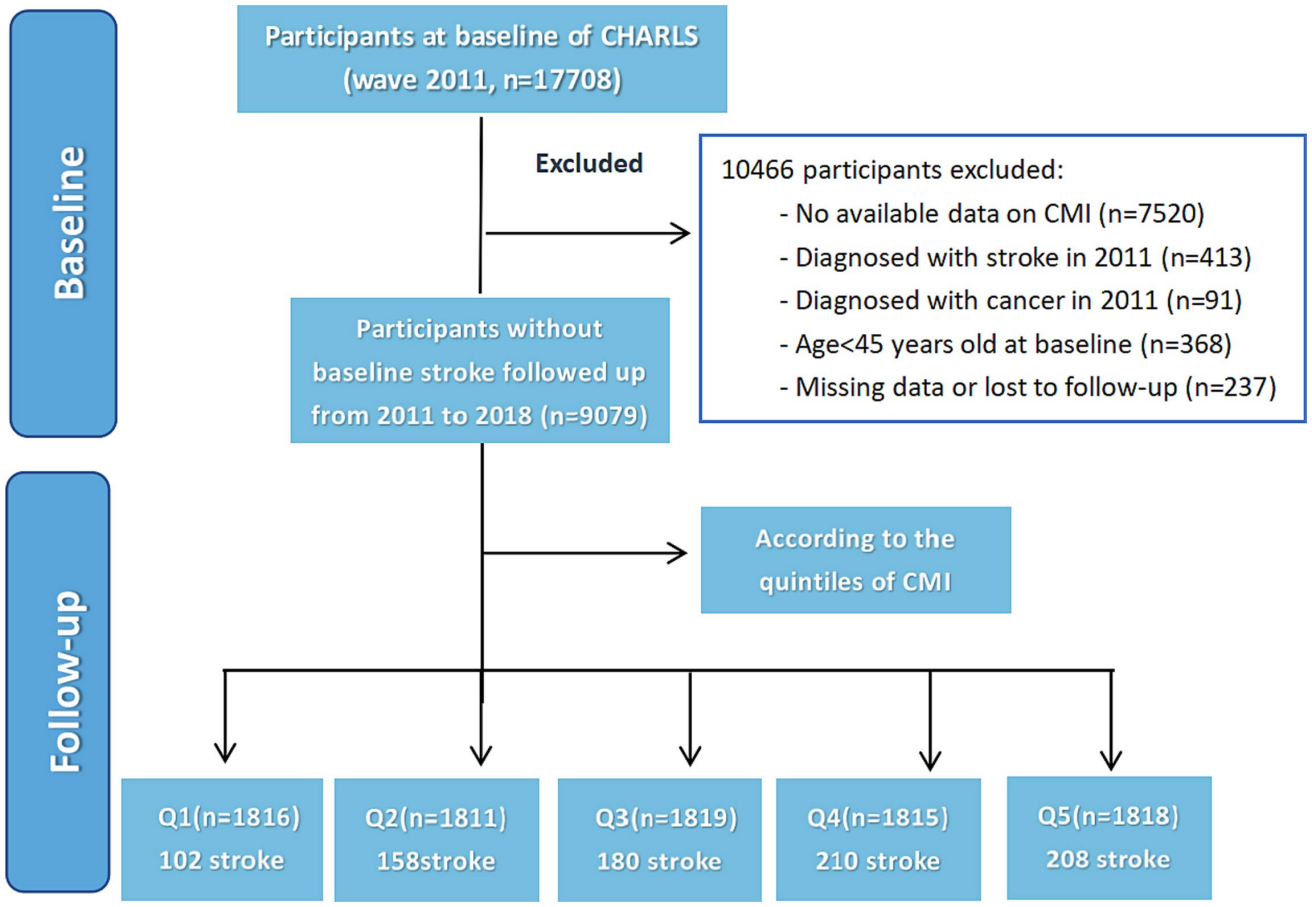
Flowchart of the study population. The CMI was categorized into five quintiles with the following ranges: Q1 (0.074–0.602), Q2 (0.602–0.935), Q3 (0.935–1.402), Q4 (1.402–2.327), and Q5 (2.327–74.326).

A total of 17,705 individuals who provided blood information in the CHARLS database 2011 baseline survey were chosen as potential study participants. Criteria for exclusion from the participant pool included individuals who suffered a stroke at baseline, with follow-up intervals shorter than 2 years, incomplete stroke data, those under the age of 45, missing baseline data for HDL-C or TG, and WHtR. Finally, 9,079 participants were enrolled, who were divided into four subgroups based on the quintiles of the CMI at baseline. The methodology and flow of participants through the study are depicted in Figure 1. This investigation corresponds to the Strengthening the Reporting of Observational Studies in Epidemiology (STROBE) publishing standards of cohort research.

### Data collection

Trained interviewers used standardized questionnaires to collect participants’ information on demographic and health-related data, while healthcare professionals conducted body measurements and laboratory tests. Data collection involved gathering a wide range of information from participants, including (1) demographic characteristics such as age, sex, height, weight, systolic and diastolic blood pressure (SBP and DBP), education level, and marital status; (2) cerebrovascular risk factors including smoking status, alcohol consumption, hypertension, diabetes mellitus and other chronic diseases; and (3) laboratory measurements like fasting blood glucose (FBG), total cholesterol (TC), TG, HDL-C, and low-density lipoprotein cholesterol (LDL-C).

### Stroke diagnosis

Stroke incidence data were systematically collected throughout the follow-up period using a structured self-reported questionnaire. The questionnaire asked participants about three key aspects: (1) Were you informed of a stroke diagnosis by a medical professional? (2) When did you initially receive or become aware of the diagnosis? (3) Do you have any therapy for your stroke at this time? Affirmative responses in subsequent follow-ups were considered as initial stroke diagnoses, with the reported date marking the onset of the condition.

To determine the timing of stroke occurrence, the interval between the reported stroke onset and the baseline assessment was calculated. For individuals who did not report a stroke during the follow-up period, the duration of their follow-up was determined by calculating the gap between the baseline evaluation and their last survey date.

### Statistical analysis

For continuous quantitative data, if the data follow a normal distribution, they are described as mean ± standard deviation, and comparisons among multiple groups are performed using one-way analysis of variance (ANOVA). If the data do not follow a normal distribution, they are presented as the median [P25, P75], and inter-group comparisons are conducted using the rank-sum test. For categorical data, they are described as frequencies (percentages), and comparisons between groups are performed using the chi-square test or Fisher’s exact test. We performed a logarithmic transformation of the CMI index and subsequently utilized the ‘rms’ package in R to construct restricted cubic spline plots to explore potential non-linear associations between the CMI index and stroke events. The median CMI index was used as the reference point, with covariates adjusted in the model. Overall association *p*-values and non-linearity *p*-values were calculated. Additionally, univariate and multivariate Cox regression analyses were conducted to investigate the relationship between CMI and stroke risk, and the hazard ratio (HR) and 95% confidence interval (CI) were determined. In these analyses, the CMI index was included both as a continuous variable and as a categorical variable grouped by quintiles with the following ranges: Q1 (0.074–0.602), Q2 (0.602–0.935), Q3 (0.935–1.402), Q4 (1.402–2.327), and Q5 (2.327–74.326). The three models included Model 1 (not adjusted for covariables), Model 2 (adjusted for age and sex), and Model 3 (adjusted for age, sex, education level, economic status, smoking, alcohol consumption, residential region, hypertension, diabetes, heart disease, and kidney disease). Cox proportional hazard models were used to independently analyze subgroups based on age, sex, body mass index (BMI), residential region, diabetes, hypertension, and heart problems to stratify the data. Trend tests based on the median value of each quintile group were conducted. To examine whether the predictive effect of the CMI index on stroke events differed by sex, the above analyses were performed separately for male and female participants. All statistical analyses and visualizations were conducted in R (version 4.4.1), with a two-sided *p*-value < 0.05 considered statistically significant.

## Results

### Baseline patient characteristics

The baseline clinical and demographic characteristics of participants categorized by CMI quintile were presented in Table 1. The average age of participants at baseline was 59.60 ± 9.36 years, with 4,212 (46.4%) being males, and 4,867 (53.6%) being female. The median CMI was 1.78 ± 2.46. Compared to those in Q1, participants in other quintiles tended to be younger; female, urban residents; have fewer current smokers and alcohol consumers; have a higher incidence of hypertension, diabetes, and heart problems; have higher waist circumference, triglycerides; and have lower HDL-C (Table 1).

**Table 1:** The baseline characteristics of participants categorized by CMI quintile.

Characteristics	Total (*n* = 9,079)	Quintile 1 (*n* = 1816)	Quintile 2 (*n* = 1811)	Quintile 3 (*n* = 1819)	Quintile 4 (*n* = 1815)	Quintile 5 (*n* = 1818)	*p*-value
Age (years)	59.60 ± 9.36	60.15 ± 9.74	59.70 ± 9.49	59.86 ± 9.32	59.30 ± 9.10	58.97 ± 9.10	0.001
Height (cm)	157.87 ± 8.55	158.33 ± 8.55	157.54 ± 8.42	157.72 ± 8.74	157.75 ± 8.58	158.01 ± 8.48	0.051
Waist circumference (cm)	85.28 ± 10.11	78.57 ± 7.91	82.17 ± 8.55	85.32 ± 9.39	88.37 ± 9.41	91.96 ± 9.47	<0.001
Triglycerides (mg/dL)	131.03 ± 92.52	59.92 ± 14.57	83.06 ± 17.63	107.50 ± 23.20	142.12 ± 30.99	262.32 ± 124.40	<0.001
HDL-C (mg/dL)	51.23 ± 15.08	68.44 ± 13.79	56.85 ± 10.83	50.57 ± 9.51	44.49 ± 8.00	35.83 ± 8.00	<0.001
CMI, Mean ± SD	1.78 ± 2.46	0.44 ± 0.11	0.76 ± 0.09	1.15 ± 0.13	1.79 ± 0.26	4.74 ± 4.25	<0.001
Gender, *n* (%)							<0.001
Male	4,212 (46.4)	1,030 (56.7)	856 (47.3)	841 (46.2)	732 (40.3)	753 (41.4)	
Female	4,867 (53.6)	786 (43.3)	955 (52.7)	978 (53.8)	1,083 (59.7)	1,065 (58.6)	
Smoke, *n* (%)							<0.001
Never	5,518 (60.8)	987 (54.4)	1,068 (59.0)	1,109 (61.0)	1,203 (66.3)	1,151 (63.3)	
Former	817 (9.0)	171 (9.4)	155 (8.6)	160 (8.8)	139 (7.7)	192 (10.6)	
Current	2,744 (30.2)	658 (36.2)	588 (32.5)	550 (30.2)	473 (26.1)	475 (26.1)	
Drinking, *n* (%)							<0.001
Never	6,223 (68.5)	1,124 (61.9)	1,242 (68.6)	1,261 (69.3)	1,306 (72.0)	1,290 (71.0)	
Seldom	1,002 (11.0)	219 (12.1)	206 (11.4)	205 (11.3)	191 (10.5)	181 (10.0)	
Regular	1854 (20.4)	473 (26.0)	363 (20.0)	353 (19.4)	318 (17.5)	347 (19.1)	
Educational, *n* (%)							0.131
Illiteracy	4,336 (47.8)	883 (48.6)	874 (48.3)	871 (47.9)	881 (48.5)	827 (45.5)	
Primary school	2076 (22.9)	426 (23.5)	424 (23.4)	427 (23.5)	390 (21.5)	409 (22.5)	
Middle school	1786 (19.7)	348 (19.2)	343 (18.9)	328 (18.0)	377 (20.8)	390 (21.5)	
High school or above	881 (9.7)	159 (8.8)	170 (9.4)	193 (10.6)	167 (9.2)	192 (10.6)	
Marital status, *n* (%)							0.066
Married	7,936 (87.4)	1,587 (87.4)	1,572 (86.8)	1,583 (87.0)	1,574 (86.7)	1,620 (89.1)	
Separated	1,080 (11.9)	208 (11.5)	227 (12.5)	227 (12.5)	231 (12.7)	187 (10.3)	
Never married	63 (0.7)	21 (1.2)	12 (0.7)	9 (0.5)	10 (0.6)	11 (0.6)	
Area Type, *n* (%)							<0.001
Rural	5,961 (65.7)	1,340 (73.8)	1,249 (69.0)	1,194 (65.6)	1,126 (62.0)	1,052 (57.9)	
Urban	3,118 (34.3)	476 (26.2)	562 (31.0)	625 (34.4)	689 (38.0)	766 (42.1)	
Hypertension, *n* (%)							<0.001
No	5,386 (59.3)	1,269 (69.9)	1,216 (67.1)	1,099 (60.4)	951 (52.4)	851 (46.8)	
Yes	3,693 (40.7)	547 (30.1)	595 (32.9)	720 (39.6)	864 (47.6)	967 (53.2)	
Diabetes, *n* (%)							<0.001
No	7,557 (83.2)	1,649 (90.8)	1,611 (89.0)	1,561 (85.8)	1,491 (82.1)	1,245 (68.5)	
Yes	1,522 (16.8)	167 (9.2)	200 (11.0)	258 (14.2)	324 (17.9)	573 (31.5)	
Heart problems, *n* (%)							<0.001
No	8,025 (88.4)	1,649 (90.8)	1,636 (90.3)	1,602 (88.1)	1,601 (88.2)	1,537 (84.5)	
Yes	1,054 (11.6)	167 (9.2)	175 (9.7)	217 (11.9)	214 (11.8)	281 (15.5)	
Kidney Disease, *n* (%)							0.285
No	8,481 (93.4)	1,682 (92.6)	1,685 (93.0)	1,697 (93.3)	1703 (93.8)	1714 (94.3)	
Yes	598 (6.6)	134 (7.4)	126 (7.0)	122 (6.7)	112 (6.2)	104 (5.7)	

CMI, cardiometabolic index; HDL-C, high-density lipoprotein cholesterol. The CMI was categorized into five quintiles with the following ranges: Q1 (0.074–0.602), Q2 (0.602–0.935), Q3 (0.935–1.402), Q4 (1.402–2.327), and Q5 (2.327–74.326).

### Associations between CMI and incident stroke

**Figure 2:**
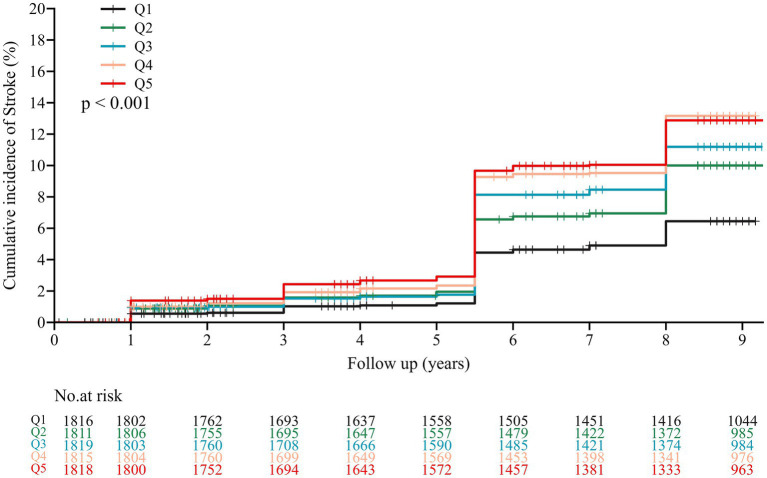
Kaplan–Meier survival curve for new-onset stroke of participants by CMI quintile. The CMI was categorized into five quintiles with the following ranges: Q1 (0.074–0.602), Q2 (0.602–0.935), Q3 (0.935–1.402), Q4 (1.402–2.327), and Q5 (2.327–74.326).

During an average follow-up period of 7.8 years, 858 (9.5%) participants experienced their first stroke. According to the CMI quintiles, the incidences of stroke for participants from Q1 to Q5, were 5.6, 8.7, 9.3, 11.6, and 11.4%, respectively. Analysis of the Kaplan–Meier cumulative incidence curve revealed a gradual increase in stroke events from the Q1 to Q5 groups in the overall population, with a statistically significant difference observed (Figure 2 log-rank test *p* < 0.001). The Cox proportional hazard models confirmed a significant relationship between baseline CMI levels and new-onset stroke. Baseline CMI was analyzed as both a continuous variable and a categorical variable (quintiles). Following adjustment for potential confounding factors, per 1-unit increase in baseline CMI was associated with a 21% higher risk of stroke in Model 3 (HR 1.21, 95% CI 1.11–1.32). Furthermore, the risk of stroke showed an increasing trend across quintiles of CMI in Model 3 (HR 1.58, 95% CI 1.23–2.03 for Q2; HR 1.67, 95% CI 1.31–2.14 for Q3; HR 1.96, 95% CI 1.54–2.49 for Q4; HR 1.76, 95% CI 1.38–2.25for Q5; *P*-trend <0.001, Table 2).

**Table 2:** The HR (95% CI) of stroke according to CMI in the three models.

Categories	Event, n (%)	HR (95%CI)		
		Model 1	Model 2	Model 3
Per-unit increase	858/9079 (9.5%)	1.31 (1.21 to 1.41) < 0.001	1.34 (1.24 to 1.45) < 0.001	1.21 (1.11 to 1.32) < 0.001
CMI quintile				
Q1	102/1816 (5.6%)	Ref.	Ref.	Ref.
Q2	158/1811 (8.7%)	1.57 (1.23 to 2.02)	1.62 (1.26 to 2.08)	1.58 (1.23 to 2.03)
Q3	180/1819 (9.9%)	1.78 (1.39 to 2.26)	1.84 (1.44 to 2.34)	1.67 (1.31 to 2.14)
Q4	210/1815 (11.6%)	2.11 (1.66 to 2.67)	2.23 (1.76 to 2.83)	1.96 (1.54 to 2.49)
Q5	208/1818 (11.4%)	2.09 (1.65 to 2.65)	2.23 (1.76 to 2.83)	1.76 (1.38 to 2.25)
*P* for trend		<0.001	<0.001	<0.001

Model 1: unadjusted. Model 2: adjusted for age and gender. Model 3: adjusted for age, sex, education level, economic status, smoking, alcohol consumption, residential region. The CMI was categorized into five quintiles with the following ranges: Q1 (0.074–0.602), Q2 (0.602–0.935), Q3 (0.935–1.402), Q4 (1.402–2.327), and Q5 (2.327–74.326).

**Figure 3:**
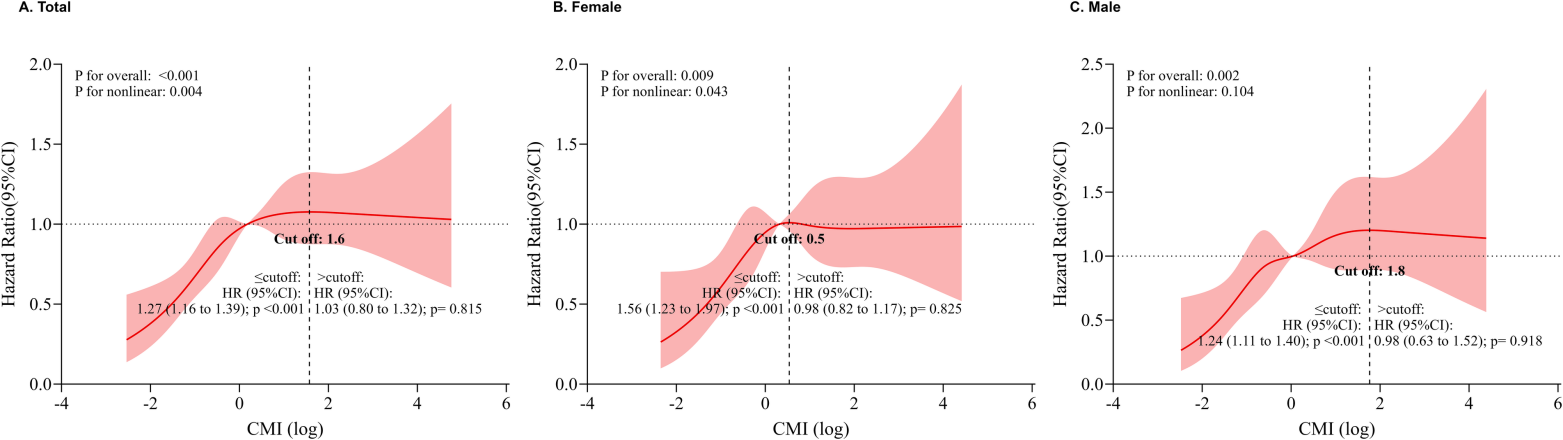
Results of RCS regression. **(A)** RCS results for the stroke. **(B)** RCS results for stroke in females. **(C)** RCS results for stroke in males. CI, Confidence interval. RCS, restricted cubic spline.

The restricted cubic spline regression analysis showed a dose–response relationship between the cumulative CMI and the risk of stroke occurrence (Figure 3). A linear correlation between the cumulative CMI and the risk of stroke occurrence in males, females, and the overall population (all *P* for overall trend < 0.01) was observed. The risk of stroke increased for individuals, with a cut-off cumulative CMI of 1.6 (HR: 1.27, 95% CI: 1.16–1.39).

### Subgroup analysis

**Figure 4:**
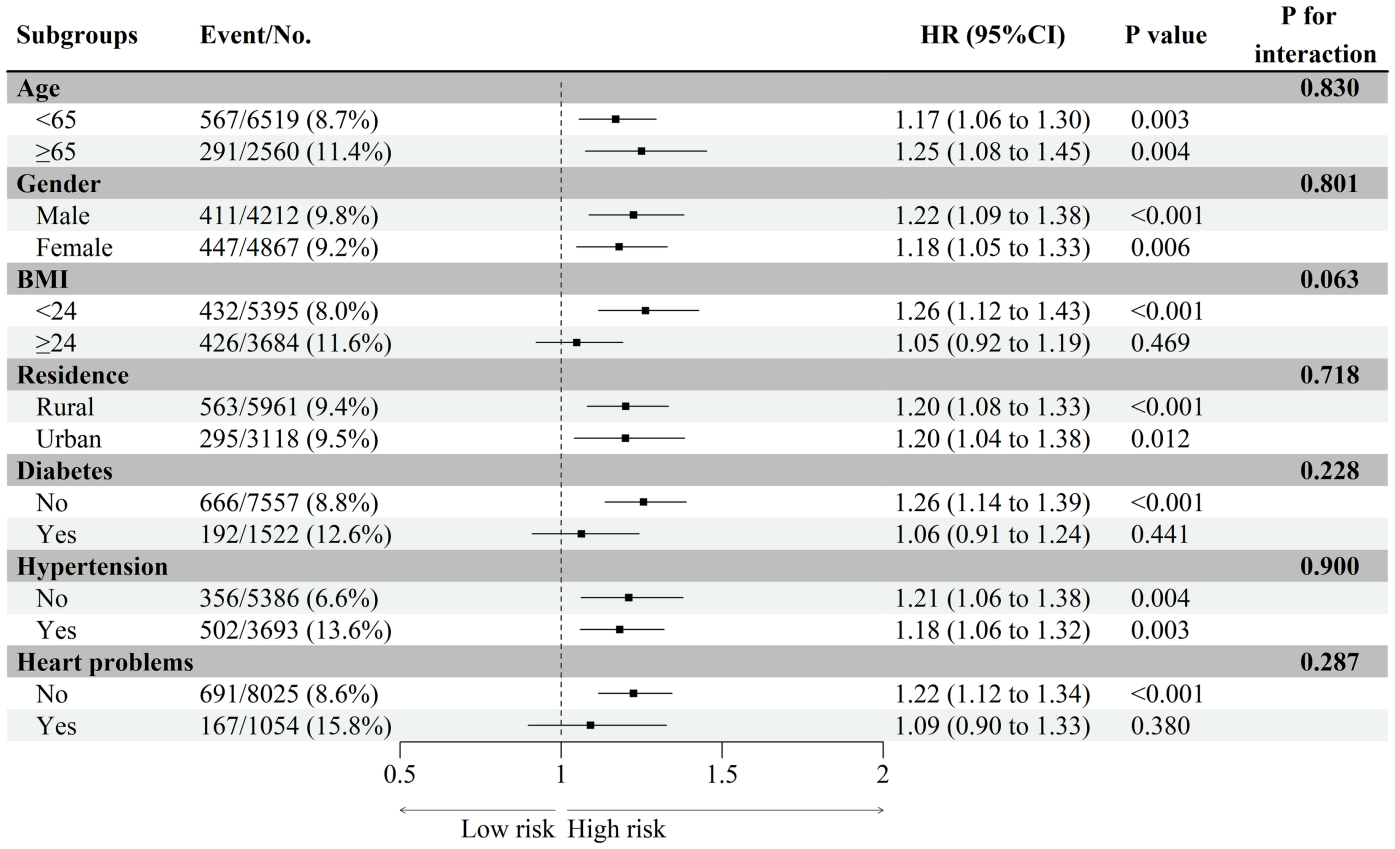
Subgroup and interaction analyses of the cumulative CMI and stroke incidence across various subgroups.

To further explore the association between baseline CMI and the first stroke event, we performed a subgroup analysis stratified by potential risk factors. As shown in Figure 4, elevated CMI levels were associated with a higher incidence of stroke, which was consistent across different subgroups including age, gender, residence, and hypertension. Among individuals with BMI < 24, without a history of diabetes or heart problems, increased CMI levels were linked to a higher stroke risk. No interaction was observed between the changes in the CMI and subgroup variables (all *p*-values > 0.05).

## Discussion

In this study, we examined the association between CMI and the risk of stroke by analyzing data from 9,079 participants from the CHARLS data. We found a significant correlation between CMI and stroke, regardless of whether CMI was assessed as a continuous or categorical variable. After accounting for several confounding factors, the results confirmed that elevated CMI values are independently associated with an increased risk of stroke. Notably, RCS analysis revealed a non-linear positive correlation between CMI and stroke prevalence, identifying a critical point at CMI = 1.6 through a threshold saturation effect. An increase in CMI within a certain range was linked to an increase in the occurrence of stroke. Subgroup analysis further showed that this correlation remained stable across various populations. These results suggest that within a specific range, CMI may be a risk factor against stroke and further research on CMI could aid in the assessment and prevention of stroke.

Cardiometabolic Index, an innovative anthropometric index, was first introduced by Wakabayashi and Daimon (15) as a tool to identify diabetes mellitus (DM) and has received growing attention since then. CMI primarily combines multiple metabolic health indicators such as body weight, blood lipids, and blood glucose to reflect an individual’s cardiovascular metabolic status (6). This index can be calculated using various methods, but the factors typically considered include: BMI, HDL-C, LDL-C, TG, blood glucose, and blood pressure. The core concept of CMI is that a single risk factor may not fully reflect an individual’s metabolic health while integrating multiple metabolic indicators can provide a more accurate assessment of cardiovascular health risks. An increase in CMI may reflect the cumulative effects of multiple metabolic abnormalities. Metabolic syndrome is one of the major risk factors for cardiovascular diseases and stroke. Abnormalities in blood pressure, blood glucose, and lipid metabolism not only increase the risk of heart disease but are also closely associated with the occurrence of stroke (16). Compared with other anthropometric and metabolic indices such as the triglyceride-glucose (TyG) index, WHtR, and atherogenic index of plasma (AIP), CMI offers distinct advantages by providing a more comprehensive assessment of cardiometabolic risk (7, 17, 18). CMI’s multidimensional nature makes it particularly useful for evaluating complex conditions such as metabolic syndrome, as it combines information on visceral fat and metabolic status for a more holistic risk stratification (7).

Previous studies have found that CMI is associated with several risk factors for stroke. For example, a close association between CMI and hypertension, diabetes mellitus, dyslipidemia, and metabolic syndrome (6, 19–21). Subsequent research further explored the association between metabolic disorders and other disease (22–27). While CMI’s predictive power is widely acknowledged, more large-scale, longitudinal cohort studies are needed to validate its applicability across diverse populations and refine its clinical thresholds for risk stratification. Data are still limited, however, on the relationship between CMI and stroke risk. A cross-sectional study revealed a positive association between CMI and stroke in the general population (28). These findings align with our results. This study, based on data from the CHARLS cohort, found a significant positive and robust correlation between CMI levels and the occurrence of stroke, with subgroup analysis yielding similar results. We also found that a higher CMI was associated with an increased risk of stroke, highlighting the importance of cardiovascular metabolic health in stroke risk assessment.

Elevated CMI reflects an interconnected network of pathophysiological mechanisms, including visceral fat accumulation, pro-inflammatory cytokine release, endothelial dysfunction, and impaired glucose and lipid metabolism. Firstly, an elevated CMI indicates an increase in visceral fat, which leads to chronic systemic inflammation (29). Several signaling pathways have been implicated in the inflammatory processes associated with elevated CMI. These include the NADPH oxidase pathway, NF-κB signaling, and T- and B-cell receptor pathways, which regulate immune cell recruitment and inflammatory responses. These pathways amplify systemic inflammation, induce endothelial dysfunction, and promote the progression of cerebrovascular diseases (30, 31). Chronic low-grade inflammation driven by elevated CMI extends its effects beyond metabolic and cardiovascular systems. It disrupts the blood–brain barrier, allowing harmful substances such as lipids and inflammatory mediators to penetrate cerebral vessels (32). Furthermore, an elevated CMI reflects the worsening of insulin resistance and lipid metabolism abnormalities, which lead to the accumulation of lipids on vascular walls, promoting atherosclerosis, vascular remodeling, and ultimately contributing to the development of hypertension (33). This increases the risk of neurovascular complications, including stroke. The role of CMI in amplifying chronic low-grade inflammation and exacerbating insulin resistance underscores its utility in identifying individuals at high risk for stroke.

### Strengths and limitations

The present study has several strengths. First, this study is a prospective cohort study, utilizing the large and representative CHARLS database. Second, the study adjusted for potential confounders and performed subgroup analyses to examine the robustness of the relationship between CMI levels and the risk of stroke in different populations. Despite this, the present study still has some limitations. First, this was an observational study. It established the associations between CMI and stroke without establishing a causative relationship. Second, although multiple confounders were considered, we could not account for all potential confounders and biases. Given that classifying stroke into subtypes could further reduce the incidence of endpoint events, we did not analyze the results based on different types of stroke. Third, the sample selected may not be fully representative of more diverse regions and populations, which may affect the generalizability of the findings. Further large-scale prospective studies and intervention trials in other countries and regions are needed.

## Conclusion

In this national prospective longitudinal study, we found a significant positive association between CMI and the risk of stroke. This suggests that CMI could serve as a promising index for improving the risk estimate of stroke in clinical practice. High CMI warranted greater attention for early risk stratification and intervention strategies in individuals at risk. Larger-scale cohort studies are needed to further validate the findings of this research.

## Data Availability

The raw data supporting the conclusions of this article will be made available by the authors, without undue reservation.

## Glossary

AIP: Atherogenic Index of Plasma
ANOVA: Analysis of Variance
BMI: Body Mass Index
CAD: Coronary Artery Disease
CHARLS: China Health and Retirement Longitudinal Study
CI: Confidence Interval
CKD: Chronic Kidney Disease
CMI: Cardiometabolic Index
CVD: Cardiovascular Disease
DBP: Diastolic Blood Pressure
DM: Diabetes Mellitus
FBG: Fasting Blood Glucose
HDL-C: High-Density Lipoprotein Cholesterol
HR: Hazard Ratio
LDL-C: Low-Density Lipoprotein Cholesterol
MetS: Metabolic Syndrome
NAFLD: Non-Alcoholic Fatty Liver Disease
NF-κB: Nuclear Factor Kappa-Light-Chain-Enhancer of Activated B Cells
RCS: Restricted Cubic Spline
SBP: Systolic Blood Pressure
SD: Standard Deviation
STROBE: Strengthening the Reporting of Observational Studies in Epidemiology
TC: Total Cholesterol
TG: Triglycerides
TyG: Triglyceride-Glucose Index
WC: Waist Circumference
WHtR: Waist-to-Height Ratio
